# SpotMetrics: An Open-Source Image-Analysis Software Plugin for Automatic Chromatophore Detection and Measurement

**DOI:** 10.3389/fphys.2017.00106

**Published:** 2017-03-01

**Authors:** Stavros P. Hadjisolomou, George El-Haddad

**Affiliations:** ^1^Department of Social and Behavioral Sciences, American University of KuwaitSalmiya, Kuwait; ^2^First Year Experience Program, American University of KuwaitSalmiya, Kuwait; ^3^Scientific Software Engineer, Scientific Software Consultancy and TrainingJeita, Lebanon

**Keywords:** chromatophore, cephalopod, image-analysis, Fiji, software, spot, SpotMetrics

## Abstract

Coleoid cephalopods (squid, octopus, and sepia) are renowned for their elaborate body patterning capabilities, which are employed for camouflage or communication. The specific chromatic appearance of a cephalopod, at any given moment, is a direct result of the combined action of their intradermal pigmented chromatophore organs and reflecting cells. Therefore, a lot can be learned about the cephalopod coloration system by video recording and analyzing the activation of individual chromatophores in time. The fact that adult cephalopods have small chromatophores, up to several hundred thousand in number, makes measurement and analysis over several seconds a difficult task. However, current advancements in videography enable high-resolution and high framerate recording, which can be used to record chromatophore activity in more detail and accuracy in both space and time domains. In turn, the additional pixel information and extra frames per video from such recordings result in large video files of several gigabytes, even when the recording spans only few minutes. We created a software plugin, “SpotMetrics,” that can automatically analyze high resolution, high framerate video of chromatophore organ activation in time. This image analysis software can track hundreds of individual chromatophores over several hundred frames to provide measurements of size and color. This software may also be used to measure differences in chromatophore activation during different behaviors which will contribute to our understanding of the cephalopod sensorimotor integration system. In addition, this software can potentially be utilized to detect numbers of round objects and size changes in time, such as eye pupil size or number of bacteria in a sample. Thus, we are making this software plugin freely available as open-source because we believe it will be of benefit to other colleagues both in the cephalopod biology field and also within other disciplines.

## Introduction

Cephalopods are renowned for their rapid body pattern change capabilities utilized in camouflage or communication (Adamo et al., 2006; Shohet et al., 2007; Hanlon et al., 2011). Sub-second body pattern transformations are enabled by the combined activity of neurally controlled intra-dermal chromatophores and reflectors (Cloney and Brocco, 1983; Sutherland et al., 2008). A lot can be learned about the sensorimotor control of body patterns by stimulating the visual system of coleoid cephalopods and measuring the resulting chromatophore activity. Since coleoid cephalopods have hundreds to thousands of tiny chromatophore organs, manual measurements and analyses of continuous chromatophore activity can become extremely complicated tasks. In this paper, we present a software plugin we developed for cephalopod researchers, “SpotMetrics,” that can automatically detect, track, and measure chromatophore activity.

### Sensorimotor control in cephalopod body patterning

Visual camouflage and signaling in coleoid cephalopods is driven by a sensorimotor system consisting of visual input of their surroundings, a sophisticated central nervous system (CNS) for information processing, and a muscular skin for generating patterns (Novicki et al., 1990). Coleoids have well-developed eyes and acute vision, which feed lobes in the CNS with input on spatial patterning, contrast, and luminance of the environment. The CNS directly controls dynamic skin changes on a massively parallel distributed system of effectors. Motor neurons selectively activate radial muscles, which in turn, produce sub-second retraction or expansion of thousands of chromatophore organs (Florey, 1969).

### Effectors

Chromatophore organs are arranged in layers with a vertical hierarchy, each layer carrying a different pigment color. For example, in the squid *Doryteuthis pealeii* (Lesueur, 1821), there are three layers: (1) the top consists of yellow chromatophore which are the smallest, (2) the middle layer with red chromatophores which are intermediate in size while, (3) the lower layer is made up of brown chromatophores which are either the same size as red chromatophores or larger (Bell et al., 2013). The organization of chromatophore layers varies between cephalopod species.

A lot can be learned about the sensorimotor control of body patterning in cephalopods by examining the chromatic changes manifested at the individual chromatophore level. This can be achieved by video recording and analyzing the expansion and contraction patterns which generate or hide colors as responses to visual stimuli. Up until now, the main difficulties that emerged from having to record and analyze individual chromatophores on cephalopods had to do with the small sizes and the vast numbers of chromatophores. For example, *Sepia officinalis* (Linnaeus, 1758) carry smaller chromatophores compared to *Doryteuthis pealeii* (Lesueur, 1821) and require a microscope or an appropriately powerful camera lens to be viewed in adequate detail. Any small movement generated by the cuttlefish may shift chromatophores outside of the point of focus. For this reason, many observations of individual chromatophores are generally done by using either dead specimens or live chromatophores on excised skin (Goodwin and Tublitz, 2013). A solution to this issue is to use high definition video standards to capture data of higher quality and use customized software to detect, track, and measure chromatophores.

### Video acquisition

Data acquisition can be improved by recording chromatophores in High Definition (HD) resolutions and at a high frame rates. Current HD commercial cameras (and even some smartphone devices) can record videos at 1,280 × 720, 1,920 × 1,080, and 2,560 × 1,440 pixel resolutions. These cameras can also record footage at 60, 120, and 240 frames per second (FPS) in NTSC format. More expensive camcorders can record at even higher resolutions (Ultra HD) and higher frame rates.

Recording at higher resolutions helps improve data acquisition, as each chromatophore is represented by more pixels and has more detail compared to lower resolutions. A higher framerate of acquisition has a shorter interval between consecutive frames and collects more images of chromatophores per second. The amount of detail per image and the rate of continuation between frames become extremely important for the task of detecting, tracking, and measuring individual chromatophores. This enables researchers to study specifics of the chromatophore sensorimotor system as a whole (for example, the pathway from eyes to brain to chromatophores) in a living animal. Therefore, higher quality video acquisition improves data collection and makes data analysis more manageable.

However, because of the additional information being stored per frame, and with more frames being recorded per second, the size of HD videos at high frame rates climbs to several GB per minute of recording. This is an important point because a larger video file will require additional processing time and computing resources which may disrupt or end altogether the normal software analysis process.

### Software to measure chromatophore activity

The following steps are necessary to extract chromatophore activity data from a series of images: (1) detect individual chromatophores and remove unnecessary background information, (2) track each chromatophore in time and space (if the footage is from an animal that moves) and, (3) measure chromatophore surface area and color. One of the main issues with analyzing individual chromatophore data has to do with the large number of objects of interest to be measured. Depending on cephalopod species and magnification level used for video recording, the observer may be looking at a few individual chromatophores on a newly hatched animal or up to a few hundred to thousands in an adult animal. Manual processing of all the chromatophores becomes extremely cumbersome, if not impossible. Therefore, an automatic system of detecting, numbering, and tracking each chromatophore (both in space and time) becomes essential for such analysis.

### Availability of software for analyzing chromatophore activity

There have been a few published studies in the cephalopod biology field which mention use of image analysis tools to measure individual chromatophore activity from video recordings (Suzuki et al., 2011; Goodwin and Tublitz, 2013; Brown, 2015; Ramirez and Oakley, 2015). These researchers either developed their own customized scripts to be used within the Matlab® software package or made use of freely available software such as Fiji (Fiji Is Just ImageJ) (Schindelin et al., 2012) and Image J (Schindelin et al., 2015). However, with the exception of a few cases, these scripts and procedures are not being made readily available online to other researchers. Any researcher interested in analyzing videos of individual chromatophore activity would have to re-invent identical methods because of lack of access to such software. In other cases, customized software uploaded on university websites may become inaccessible during site server changes.

These issues can be prevented by following software development guidelines on sharing code and data and properly documenting the software on repository sites such as GitHub or SourceForge. Another factor which limits accessibility to software has to do with licenses and limited installations of paid-for applications. Commercial software solutions can be very powerful for data collection or analysis and are more likely to be continuously supported by dedicated developer teams compared to free open sourced software. However, the high cost per license may be prohibitive for students without sufficient funding. As a solution to this problem, students can choose to use free software, so the idea of developing and publishing programs on open-source platforms is well worth pursuing. Research can be accelerated by having methods and data publicly accessible to any scientist who may benefit from it. For this reason, we designed and created a software plug-in, SpotMetrics, that can analyze and process large HD video files in a freely available image analysis program.

### SpotMetrics

The main objective of SpotMetrics is to process and analyze large HD video recordings of chromatophores. The plug-in automatically detects and numbers individual chromatophores, tracks them for the duration of the video, and provides information about their surface area and color properties. We would like to emphasize here that such a software solution has been published (Goodwin and Tublitz, 2013) as a customized script to be run within the Matlab® software package. The differences between this publication and Goodwin and Tublitz (2013) is that SpotMetrics is based on completely free and open-source software. Also, we are expanding on the ideas presented in that paper by adding a system that can track hundreds of chromatophores moving within the 2D space domain. We are working with a freely available software, Fiji, which does not require a license purchase for usage since it is under General Public License (GPL). In the same manner, we are making SpotMetrics software available to all under a GPL. Also, we are welcoming others to contribute to this software by contacting us with suggestions and requests to be worked into the existing plugin. Additionally, the plugin will be uploaded and maintained on a GitHub server which will contain periodic updates.

## Methods

SpotMetrics is a software plugin developed in JAVA specifically to be used with the image analysis suite Fiji.

Fiji is a suite of plugins and most of those plugins are a collection of algorithms themselves. The main reason that Fiji is so successful in the scientific community is that it allows the combination and reuse of other software and algorithms.

### Libraries

SpotMetrics makes use of the following libraries which are used to detect, track, and measure the spot properties within a particular video:

*AVI_Reader* (https://imagej.nih.gov/ij/plugins/avi-reader.html) is used to import and read video files (.avi format) of chromatophores or other objects of interest to be analyzed.
*TrackMate* (http://imagej.net/TrackMate) detects and tracks every spot for the entire length of the video. Also, TrackMate keeps a record of every spot's X, Y coordinates from every frame for later reference. Lastly, it allows users to filter out spots that are of no interest.
*Particle Analyzer* (http://imagej.net/Particle_Analysis) analyzes and keeps track of the Regions of Interest (ROIs) in each frame and measures the area of each spot.
*Measure Color* (author: George El Haddad) is a simple algorithm that retrieves the central X, Y coordinate of each spot (with the help of TrackMate) and return the Red, Green, and Blue (RGB) values for the spot.
*Apache POI* (https://poi.apache.org/) is a fast and highly scalable library used to export data to a Microsoft Excel file (.xlsx). It is capable of generating large Excel files, depending on the amount of data to be exported.

### Protocol

The following steps outline the procedure used by SpotMetrics to filter images and enhance chromatophore detection, tracking, and measurement.

#### SpotMetrics procedure for analyzing spots/chromatophores in videos

1. Image processing and background subtraction:
  - Program processes the imported video by converting all frames to 8-bit gray scale.
  - Uses Fiji's Auto Threshold on every frame (method = “Default”) to remove background and keep spots (chromatophores) for further analysis.
2. Spot detection and tracking:
  - Analyze objects in image to identify/detect spots in each frame.
  - Based on user settings (spot size), filters out spots that do not fit in selected category.
  - Each spot is identified by a unique number.
  - Track spots of interest over the length of video to collect data on size and color.
3. Data output.
  - The program will generate an Excel file containing two spreadsheets with size and color measurements for further analysis:
    - First spreadsheet lists the surface area (in pixels squared) of each spot per each frame.
    - Second spreadsheet lists the RGB values of each spot per each frame.

#### Step-by-step instructions to run SpotMetrics analysis

For first-time users, we recommend to run the program using the default options initially and to adjust settings for subsequent analyses based on results.

**Figure 1:**
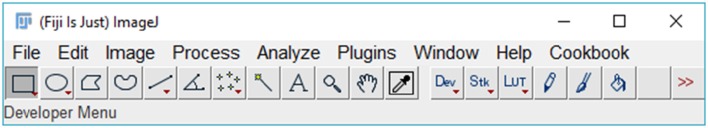
**Main menu of Fiji software**.

**Figure 2:**
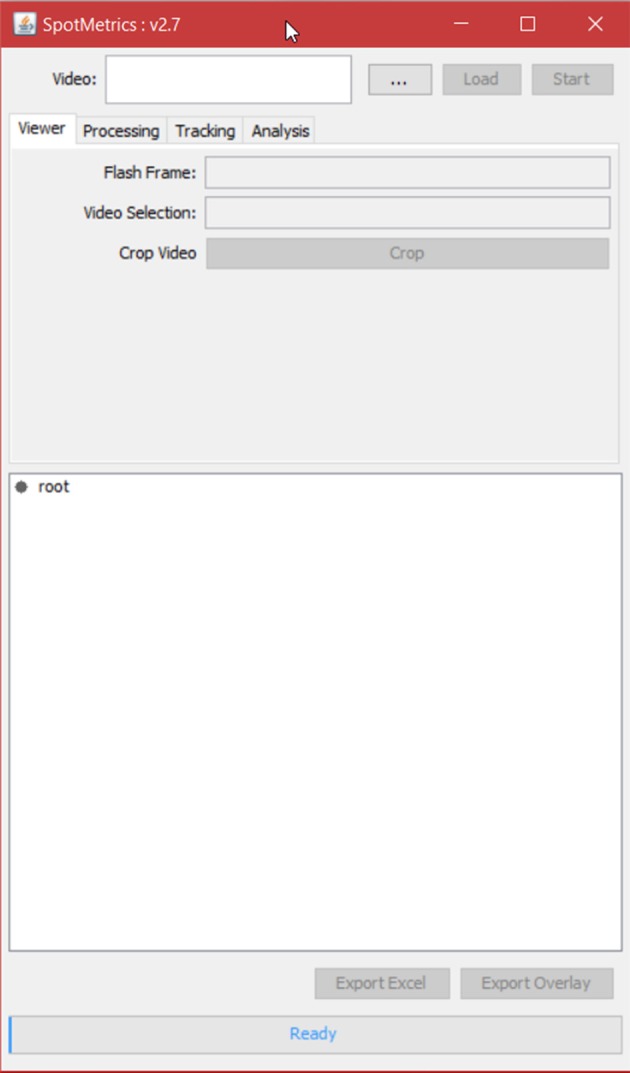
**Main menu of SpotMetrics**.

**Figure 3:**
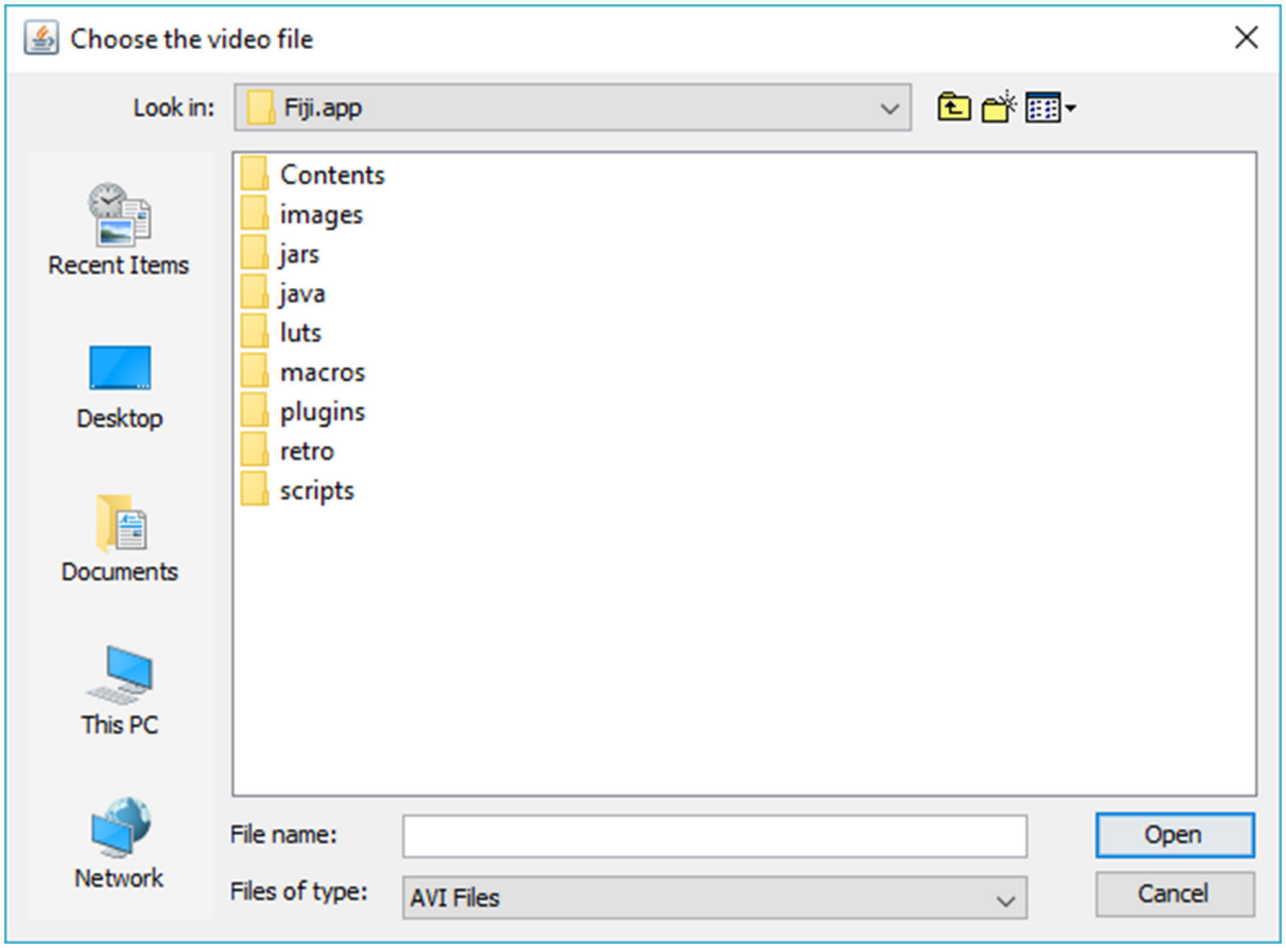
**Browse to desired directory to load video file**.

1. Run Fiji (Figure 1).
2. Click on “Plugins” menu and select “SpotMetrics.”
3. Click “…” button and browse to folder containing the video you want to import. Choose video and click “Open” (Figures 2, 3).

Note on video file format compatibility: The user must first convert all video files to be used with SpotMetrics into the.avi format. This is a requirement of the AVI_Reader library that is used here to import video files: “*PC users can use the free VirtualDub program to uncompress AVI files. Macintosh users can use QuickTime Pro to convert QuickTime movies into uncompressed AVI movies. Note that AVI files with audio tracks may fail to open*.” (https://imagej.nih.gov/ij/plugins/avi-reader.html).

**Figure 4:**
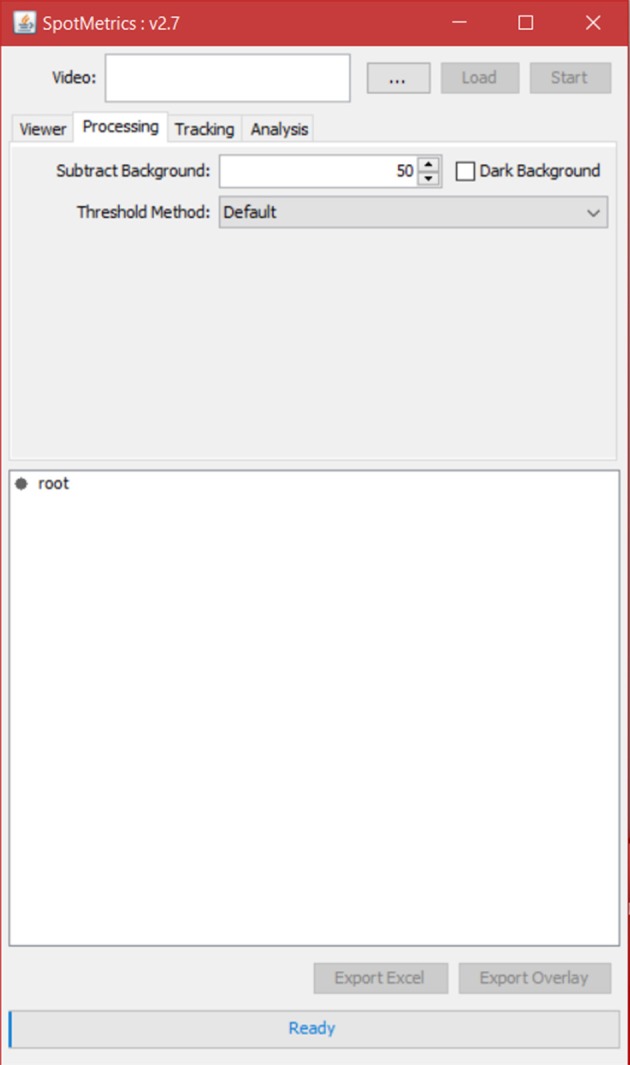
**Processing tab. Default settings will remove light background and keep dark objects**.

**Figure 5:**
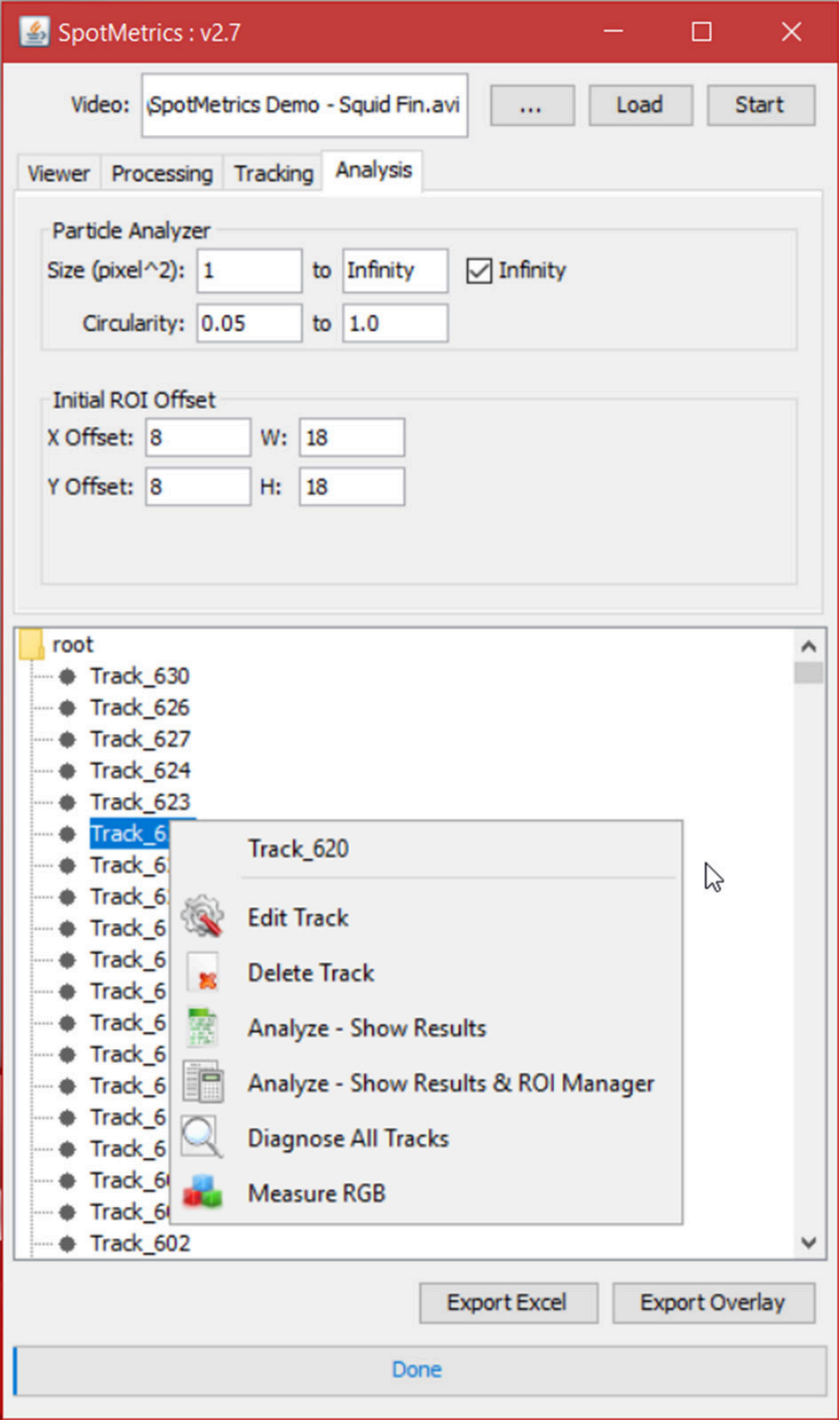
**Tracking tab**. Choose blob diameter in pixels to track chromatophores of such diameter. Choose threshold value to exclude all chromatophores with diameters smaller than threshold value.

**Figure 6:**
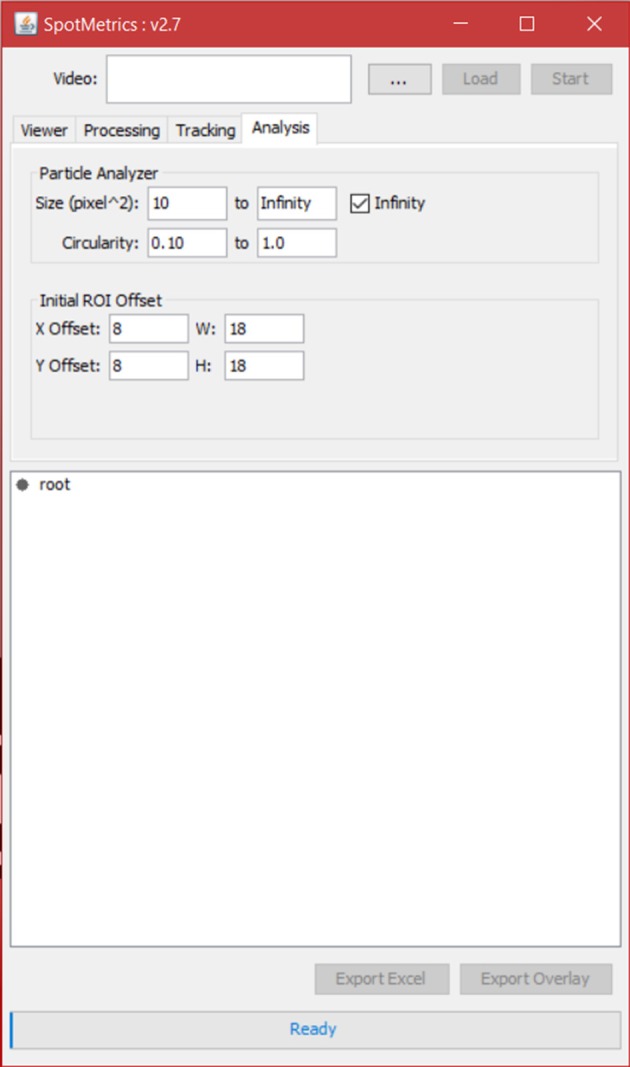
**Analysis tab**. Set Search Region dimensions (ROI offset).

4. The user has the option to crop part of the video at this point by using the Fiji available editing tools.
5. Click on the “Processing” tab to set video processing options (Figure 4).
  - Keep the default settings for the initial run. The default settings will be “subtract background: 50” and “dark background” unchecked. Threshold method will be set to “default.”
6. Click on the “Tracking” tab to set the spot tracking options (Figure 5).
  - All measurements are in pixels, so set the “blob diameter” to a value close to each spot. For example, the default blob diameter is set to “10” pixels.
  - The “blob threshold” value. Any chromatophore smaller than this diameter will not be tracked. Thus, if the user is only interested on larger spots, then a lot of the smaller ones can be filtered out using this setting.
  - For the remaining options, we recommend leaving the default values as is:
    - Linking max distance set to “15”
    - Gap closing max distance set to “15”
    - Gap closing max frame gap set to “2”
    - Initial spot filter value set to “0”
7. Click on the “Analysis” tab to set particle analysis options (Figure 6).
  - Set the square area of the particle to detect (this is in pixels squared).
  - Set the circularity of the particle being detected where 1.0 is a perfect circle.
8. Set the initial ROI offset for each spot that the particle analyzer will scan inside of. This is the initial box that will be drawn around each spot; the particle analyzer will scan inside this box so this has to be more or less accurate. If not, it can be tweaked later.
9. Click “Start” to initiate analysis.

#### Post-analysis steps to improve measurements manually

Once the program has analyzed the video and measured spot properties, the user can run diagnostic tests to see how many spots have missing data and apply edits to fix the issues.

**Figure 7:**
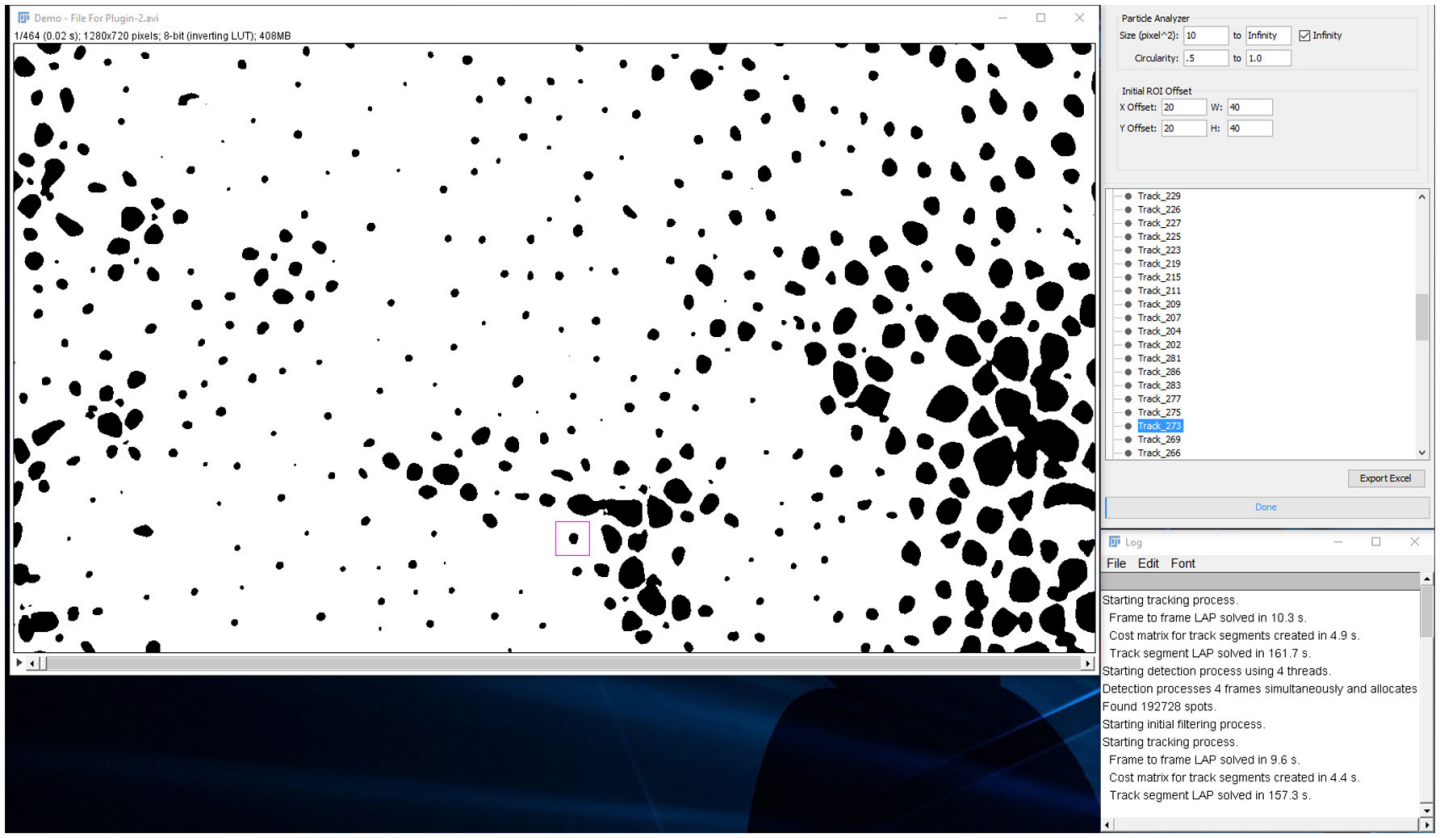
**Analysis tab**. Results tree with individual chromatophores being represented as tracks.

**Figure 8:**
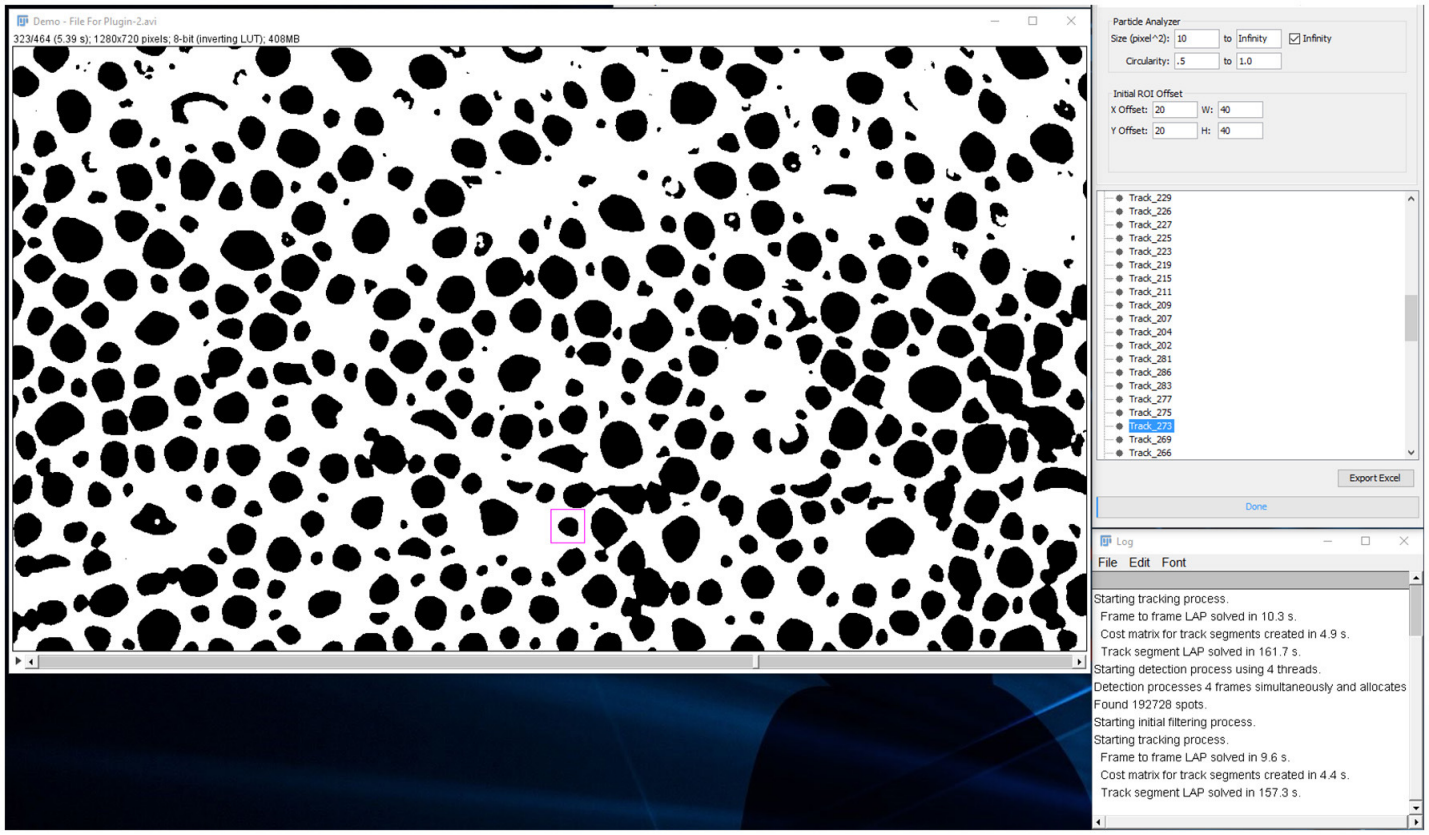
**Analysis tab**. Users can playback the video and inspect individual tracks as chromatophores expand or retract.

When analysis is done and the spots are listed in a tree menu: (Figures 7, 8).

1. In the tree menu, right click on any track and select menu item “Diagnose All Tracks”
  - This instructs the plugin to perform a particle analysis based on the “Particle Analyzer” options under the “Analysis” tab.
2. The “scan results” report indicates:
  - Number of spots per track.
  - Number of tracks with missing spots.

From this point in analysis, the user can manually edit each track that has missing spot measurements in specific frames.

1. Right click and select menu item “Edit Track”
  - The ROI around the spot will turn from magenta to blue to indicate it is being edited.
  - Click the “Next” button to scan frames forward to find the spot that is not being detected by the particle analyzer.
  - Normally the issue is that the spot touches the ROI boundary, either because the is ROI is too small or slightly offset.
  - Adjust the blue ROI with the mouse so that it properly encapsulates the spot and has at least a one-pixel gap between.
  - Click “Update ROI.”
  - Continue until all frames are accounted for then close the “Edit Track” pop-up window.
2. Right click and select menu item “Analyze—Show Results.”
  - Make sure that all the spots are properly detected and there is no missing information from any frames.

**Figure 9:**
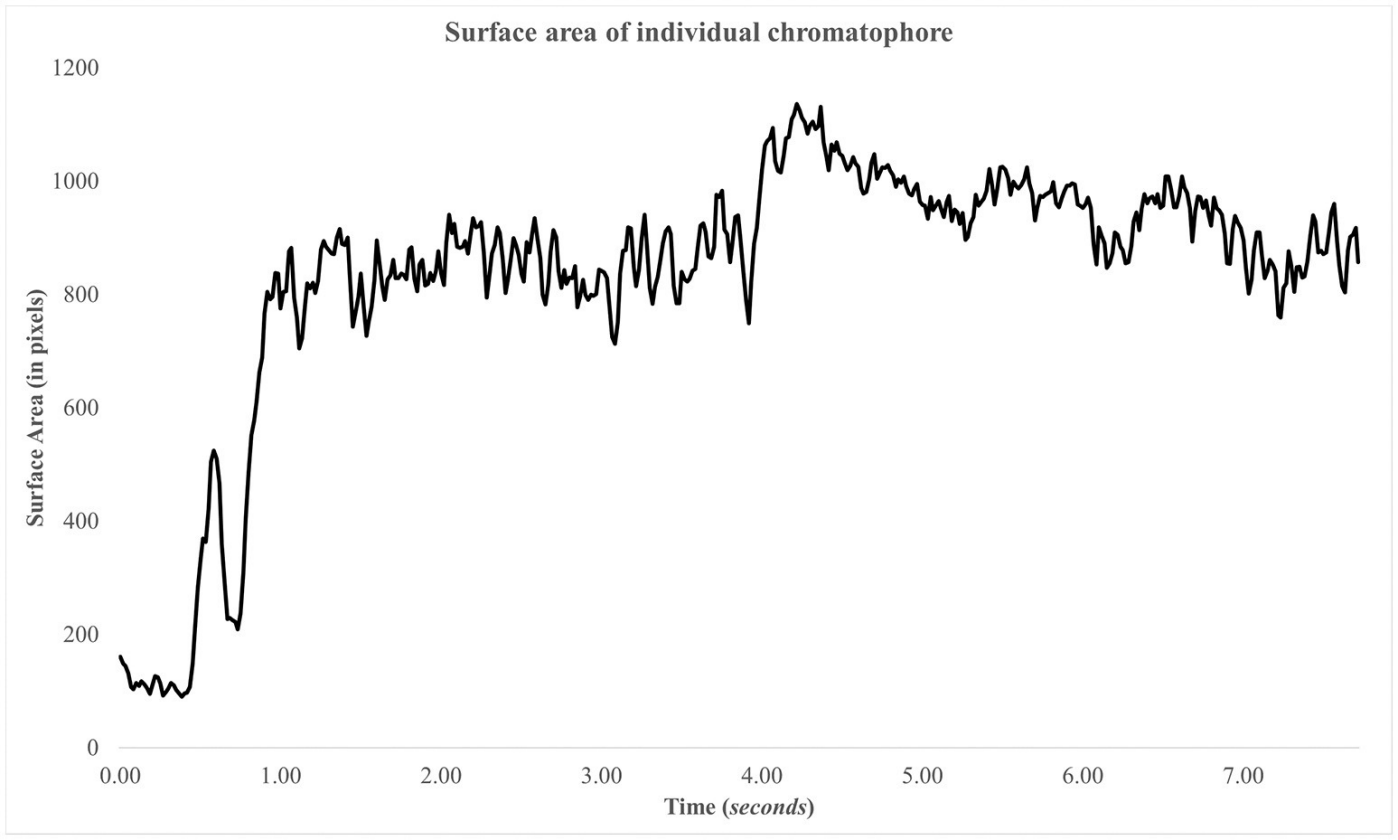
**Surface area of chromatophore over time**. The chromatophore expands rapidly within the first second and remains expanded. There's another expansion, minor, at ~4 s.

**Figure 10:**
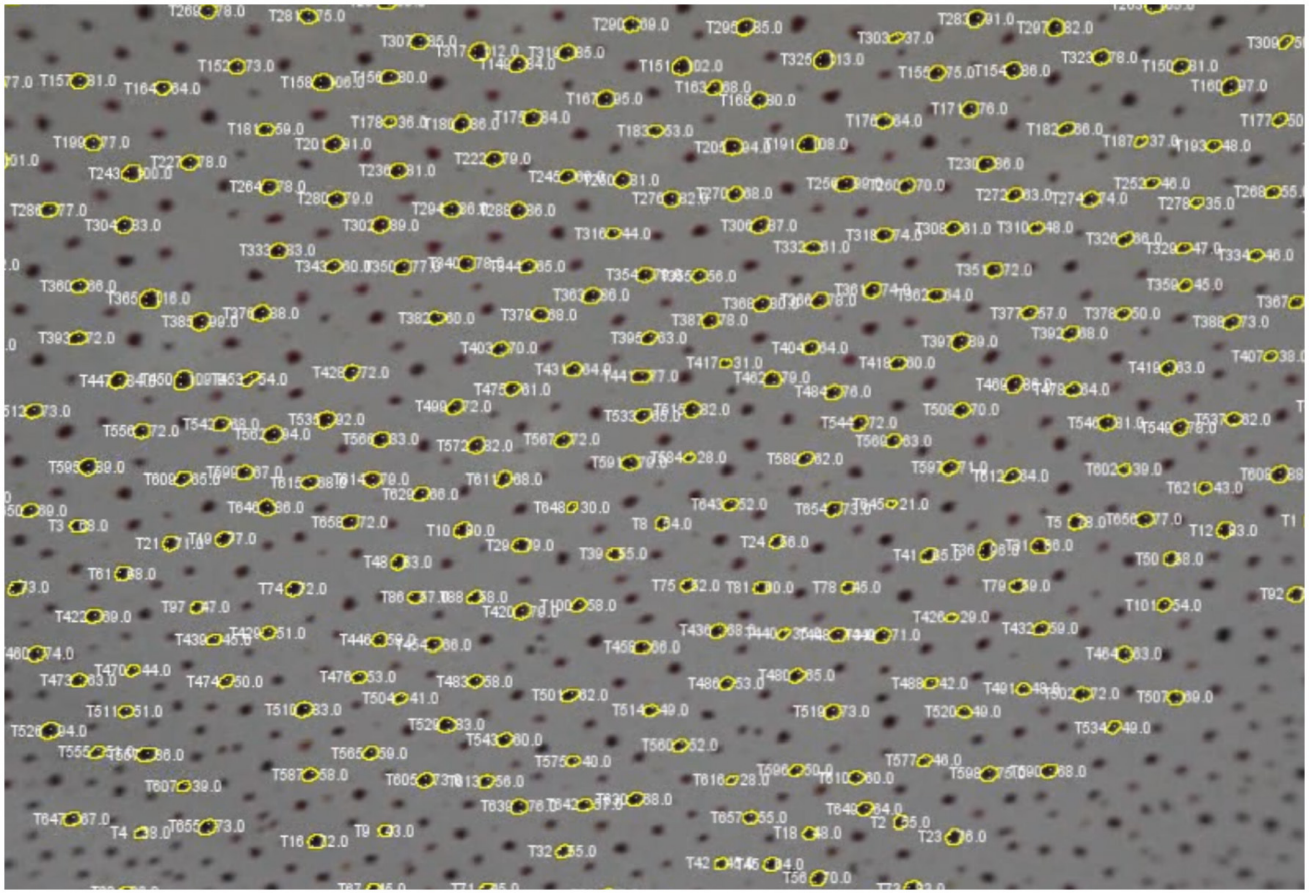
**Screenshot of video with each tracked chromatophore and corresponding outlines as overlays**.

Also, the user can delete any track from the tree menu:

3. Right click and select menu item “Delete Track.”
4. Click the “Export Excel” button and choose a directory and filename to save the chromatophore data in an Excel sheet. You can create graphs to visually inspect the chromatophore activity (Figure 9).
5. Click on the “Export Overlay” button to render a new video in which each tracked spot is shown with its outline as an overlay (Figure 10). Each spot is labeled based on two things: (1) the numbered ID of the track, and (2) the surface area measurement in pixels for each track. This is a valuable feature for reviewing each analysis, either to detect any errors, or to visually investigate each tracked chromatophore activity compared to others in time. This video can be saved for presentation purposes (Videos 1 and 2).

## Limitations and considerations

### System requirements

SpotMetrics makes heavy use of CPU and RAM resources which are reallocated to speed up the analysis. The processing time of SpotMetrics is estimated by the number of chromatophores/spots that are detected within a given video file. If the plugin has to analyze 100,000 spots, the processing time will be significantly longer than analyzing 1,000 spots. We highly recommend using a PC with a modern processor to run this plugin. In addition, users should avoid having other utilities run in the background while SpotMetrics is processing data.

### Image quality

The success of this plugin depends heavily on the image quality of the imported video files. For example, chromatophores/spots that appear out-of-focus/blurry will be more difficult to be detected and tracked by the plugin. In turn, the plugin will extract less information from such videos compared to recordings in which chromatophores/spots appear in more detail (Figure 9).

In addition, shaky footage, or lots of movement of chromatophores in the video can decrease the efficiency of detection and tracking over time. If the chromatophores/spots are displaced in the 2D domain rapidly, then the program will have difficulty tracking them for the entirety of the video. However, this can be corrected, up to a point, by applying a stabilization tool over the original video, using popular video editing software such as Sony Vegas Pro or Adobe After Effects. Nevertheless, we advise to proceed with caution whenever using video effects in this manner as to avoid modifying the recorded video in ways which may alter the image and add artifacts.

### Possible chromatophore omission due to top-down perspective of camera angle

Cephalopod chromatophores are stacked in a vertical hierarchy (see section Effectors). Depending on the species, the top layer consists of either the largest chromatophore type (such as in some octopus species) or the smallest (such as in some squid species). The specific chromatophore arrangement becomes important when considering SpotMetrics detects and tracks objects within a 2D image; if two objects in a 3D environment are situated in such a way that the larger object is directly above a smaller object, then when converting the image to 2D, the larger object will appear to cover the one below when viewed from a top-down perspective. In this case, the smaller object below is not available for observation and thus SpotMetrics will not detect it.

When examining cephalopod body patterning, if a video sequence consists of fully expanded chromatophores within the top layer of Octopus skin, and given this type of chromatophores is the largest, then the smaller chromatophores below could be completely covered and hidden from observation from a top-down perspective. In this example, SpotMetrics would not be able to detect the hidden chromatophores, and would instead detect, track, and measure only the top layer of chromatophores. Therefore, to prevent this ommission when studying the chromatophore activity from a top-down perspective, it's vital to consider this fact to choose an appropriate choice of cephalopod species to study.

## Significance of SpotMetrics as a research tool

SpotMetrics' value as a research tool comes in the form of automation and simplification for the researcher. This plugin would take several days' worth of manual processing down to an hour with just a few clicks in a user-friendly graphical environment. It does so by utilizing the power of existing algorithms, tools, and libraries provided by the scientific imaging open-source community and combining them for the use of tracking and analyzing spots.

Without a plugin such as SpotMetrics, a researcher would have to use a different plugin for each of the following steps: (1) to filter video data based on the contrast of the image and to remove unnecessary background information, (2) to identify and use the right method to detect spots in a video, (3) track spots over a duration of time and through space, (4) to analyze the spots that have been tracked for their color properties, (5) to analyze spots for surface area changes, and (6) to extract and collect the data from each individual tool and consolidate them into an organized spreadsheet ready for statistical analyses of results. Undertaking each of the above steps manually is technically possible. However, in this scenario, the researcher would have to spend a considerable amount of time on research, and trial-and-error process, in getting the output from each plugin to be formatted appropriately to be used as input for the next step. SpotMetrics automates all the above processes, saving the researcher valuable time which would have otherwise been spent on researching and troubleshooting each method.

## Applications and conclusions

SpotMetrics can be used in a variety of image analysis procedures with the goal of detecting, tracking, and measuring circular objects over a period of time. This plugin was developed with the primary goal to contribute to existing studies on sensorimotor integration control of the chromatophore system in cephalopods. We believe this tool will be helpful to researchers who wish to examine chromatophore activity as a response to sensory stimulation to better understand the underlying mechanisms of both sensors and effectors. For example, SpotMetrics can be used to measure chromatophore responses when animals are tested behaviorally under different chemical agents, presented with different background information, or presented with a potential predator, prey, mate, or competitor.

Similar to cephalopod biology studies, SpotMetrics can be of benefit to researchers who study chromatophore and melanophore systems in fish and reptiles. Lastly, this software can potentially be utilized to detect numbers of round objects and size changes in time, such as pupil dilation studies or number of bacteria in a sample.

We are making SpotMetrics freely available under the GPL license. The source code will be made available on the GitHub repository which will enable easier access to the latest update of the program. We'd like to extend an invitation to interested parties who may want to collaborate on improving this plugin by adding customized features and expanding the scope of the software in analyzing experimental data.

## Ethics statement

The animals used in the present study were squid. There was no requirement for this study to be approved by an ethical committee since at the time this study took place (July, 2014) the Institutional Animal Care and Use Committee (IACUC) protocols were not issued for invertebrate research in the USA. Nevertheless, we took every precaution to ensure the animals were under the least amount of stress possible and were not harmed in this study. Furthermore, the animals were allowed to acclimate when transported from housing to the experimental rig and vice versa. The study was observational and the animals were not subjected to any invasive methods.

## Author contributions

SH contributed to the conception and design of the work. SH contributed to the drafting the manuscript. SH gave final approval for this version to be published. SH agrees to be accountable of all aspects of work in ensuring that questions related to the accuracy or integrity of any part of the work are appropriately investigated and resolved. GE contributed to the software development presented in this paper. GE contributed to revising the manuscript. GE gave final approval for this version to be published. GE agrees to be accountable of all aspects of work in ensuring that questions related to the accuracy or integrity of any part of the work are appropriately investigated and resolved.

## Funding

The study was supported by a doctoral student research grant from the Graduate Center of the City University of New York to SH.

### Conflict of interest statement

The authors declare that the research was conducted in the absence of any commercial or financial relationships that could be construed as a potential conflict of interest. The reviewer JJC and handling Editor declared their shared affiliation, and the handling Editor states that the process nevertheless met the standards of a fair and objective review.
